# PCAF Accelerates Vascular Senescence via the Hippo Signaling Pathway

**DOI:** 10.1155/2022/1200602

**Published:** 2022-10-06

**Authors:** Chaohua Kong, Dongchen Wang, Feng Wang, Yifei Lv, Wenying Zhou, Peng Ye, Yue Gu, Xiaomin Jiang, Linlin Zhu, Zhen Ge, Yuelin Chao, Shaoliang Chen

**Affiliations:** Department of Cardiology, Nanjing First Hospital, Nanjing Medical University, Nanjing, China

## Abstract

P300/CBP-Associated Factor (PCAF), one of the histone acetyltransferases (HATs), is known to be involved in cell growth and/or differentiation. PCAF is reported to be involved in atherosclerotic plaques and neointimal formation. However, its role in cellular senescence remains undefined. We investigated the potential mechanism for PCAF-mediated cellular senescence. Immunohistochemical (IHC) analysis showed PCAF was distinctly increased in the endothelia of aorta in aged mice. Palmitate acid (PA) or X radiation significantly induced the expression of senescence-associated markers and PCAF in human umbilical vein endothelial cells (HUVECs). PCAF silence in PA-treated HUVECs significantly rescued senescence-associated phenotypes, while PCAF overexpression accelerated it. Additionally, our results showed that Yes1 Associated Transcriptional Regulator (YAP) that acts as end effector of the Hippo signaling pathway is crucial in PCAF-mediated endothelial senescence. YAP activity declining was observed in aged vascular endothelia. Overexpression of YAP partially ameliorated PCAF-induced endothelial senescence. In vivo, endothelial-(EC-) specific PCAF downregulation in aged mice using adeno-associated virus revealed less vascular senescence-associated phenotypes. These results suggested that PCAF mediated endothelial senescence through the Hippo signaling pathway, implying that PCAF may become a potential target for the prevention and treatment of vascular aging.

## 1. Introduction

Cellular senescence is an irreversible form of cell cycle arrest that cells lose their replicative capacity and halt cell cycle in the G1 and G2 phases [1]. It is evoked during embryonic development as well as by various stressors, including oxidative stress, inflammation, UV and/or ionizing radiation, chemotherapeutic agents, aberrant activation of oncogenes, and inactivation of tumor suppressor genes [2]. Cellular senescence influences organic senescence, result in a series of disorders like obesity, cardiovascular diseases, diabetes, and neurodegeneration.

Endothelial cell is one of the main cell types that constitutes the vascular system. Senescent endothelial cells exhibit impaired homeostatic functions including reduced nitric oxide production and increased generation and secretion of ROS and a wide range of cytokines and chemokines like interleukin (IL)-1*β* and interleukin (IL)-6 [3–6]. Accumulating evidence suggests endothelial senescence renders the vessels prone to profound functional and morphological disturbances that ultimately bring about various cardiovascular diseases. Thus, understanding the mechanism of endothelial senescence is critical to prevent senescence-associated cardiovascular disease. Several senescence drivers and relevant pathways have been summarized to be associated with senescence establishment, including two classical tumor suppressor pathways, p53/p21 and Rb/p16 [7]. However, the specific molecular mechanisms remain poorly elucidated.

P300/CBP-Associated Factor (PCAF), a member of the GCN5-related N-acetyltransferase family, functions as a histone acetyltransferase (HAT) to promote transcriptional activity [8]. Apart from its role in acetylating core histones (H3 and H4), it can also interact with many nonhistone proteins. PCAF has been shown to participate in the modulation of arteriogenesis, cell cycle progression, differentiation, gluconeogenesis, and tumorigenesis [9–11]. Intriguingly, several studies indicate that PCAF is implicated in the activation of p53-dependent transcription of the cyclin-dependent kinase inhibitor p21 and p16, which are highly associated with senescence establishment [12, 13]. Therefore, we hypothesized that inhibition of PCAF in endothelial cell may attenuate cellular senescence.

The aim of this study was to reveal the underlying molecular mechanism by which PCAF contributes to cellular senescence. By using PA-induced cellular senescence and aged mouse model [14, 15], we identified a novel and unrecognized role for PCAF in promoting cellular senescence through the Hippo signaling pathway. Our findings indicated that PCAF may become a promising therapeutic target for senescence-associate vascular disease.

## 2. Materials and Methods

### 2.1. Animal Tissues and Experiments

All experimental protocols and animal use were approved by the Institutional Animal Care and Use Committee of Nanjing Medical University. One and half-year-old and 4w C57BL/6 male wild-type mice (*n* = 21) were purchased from the Model Animal Research Centre of Nanjing University. Mice were housed in a specific pathogen-free room with an ambient temperature of 25°C and a humidity between 30% and 70%. They were exposed to 12-h light–dark cycles and fed with rodent food and adequate water. All animals were allocated randomly into three groups based on a single sequence of random assignments. Aortic arteries were dissected and fixed in 4% paraformaldehyde overnight at 25°C, and then embedded in paraffin.

### 2.2. Cell Culture

Human umbilical vein endothelial cells (HUVECs; Cellbank of Chinese Academy of Sciences, Shanghai) were cultured at 37°C in a humidified 5% CO_2_ incubator. Cells were cultured with endothelial culture medium (ECM) with 5% fetal bovine serum and 1% penicillin-streptomycin (Sciencell, USA). When the cells reached 80–90% confluence, they were trypsinized and subcultured.

### 2.3. Materials and Reagents

Primary antibody against PCAF (3305, CST), p53 (sc-126, santa cruz), p21 (sc-6246, santa cruz), p16 (sc-166760, santa cruz), Ubiquitin (sc-53509, santa cruz), phosphor-Histone H2A.X(Ser139) (#2577, CST), YAP (#14074, CST), MDM2 (BS-1223, Biogot), phospho-YAP (#13008, CST), TAZ (#83669, CST), phospho-TAZ (#59971, CST), clathrin (ab21679, abcam), caveolae (ab2910, abcam), mTOR (ab2732, abcam), p-mTOR (Ser2448) (ab109268, abcam), p-mTOR(Ser2481) (ab137133, abcam), IL-1*β* (sc-12742, snata cruz), IL-6 (sc-130326, santa cruz), GAPDH (#AP0063, Biogot), histone H3(BS3718, Biogot), and PA (P0500, Sigma-Aldrich) were purchased commercially.

### 2.4. Western Blotting

Cells were lysed in a mixture of RIPA and proteinase inhibitor (100 : 1) for 20 min prior to centrifugation at 15 000 × *g* for 15 min at 4°C, as described in the previous studies. Protein concentration was determined by a bicinchoninic acid protein assay. Protein lysates were boiled in sodium dodecyl sulfate (SDS) sample buffer at 94°C for 7 min. 40 *μ*g proteins were resolved on 10% sodium dodecyl sulfate-polyacrylamide gels, and then transferred to PVDF membrane. After that, the membranes were blocked in 1 × TBST containing 5% skim milk for 1 hour at room temperature before incubated with indicated primary antibodies at 4°C overnight.

### 2.5. Quantitative Real-Time PCR

Total RNA was isolated using Trizol reagent (Vazyme, R401-01, Shanghai, China) according to the manufactures' protocols [16]. RNA was reverse transcribed using HiScript II Q Select RT SuperMix (Vazyme, Shanghai, China) for qPCR and qPCR was performed on a Fast 7500 cycler (Applied Biosystems) using the resultant cDNA along with Taq Pro Universal SYBR qPCR Master Mix (Vazyme, R232-01, Shanghai, China) and gene-specific primers. PCR primers used are listed as follows:

IL-1*β*: Froward: ATGATGGCTTATTACAGTGGCAA.

Reverse: GTCGGAGATTCGTAGCTGGA.

IL-6: Froward: ACTCACCTCTTCAGAACGAATTG.

Reverse: CCATCTTTGGAAGGTTCAGGTTG.

GAPDH: Forward: GGAGCGAGATCCCTCCAAAAT.

Reverse: GGCTGTTGTCATACTTCTCATGG.

PCAF: Forward: CGAATCGCCGTGAAGAAAGC.

Reverse: CTTGCAGGCGGAGTACACT.

Results were qualified using a delta-delta-cycle threshold (Ct) method (ΔΔCt). All experiments were performed in triplicate and GAPDH was used as an internal control.

### 2.6. Cell Transfection

HUVECs were transfected with 3.75 *μ*l PCAF/YAP or negative control siRNA using Lipo3000 after cell confluence reached 70–80% according to the manufactures' protocols. When cell confluence was 90%, 2.5 ng plasmid and corresponding control plasmid along with 3.75 *μ*l Lipo3000 and p3000 (L3000-015, Thermo Fisher) were cocultured with HUVECs in opti-MEMI medium (Gibco,31985070) for 6 hours then refreshed with ECM. Cell lysates were collected after 48 h infection.

siRNA was purchased from Gene Pharma (Shanghai, China) siRNA targeting

PCAF#1: AGAGCAGUCCUGGAUUA,

PCAF#2: UCGCCGUGAAGAAAGCGCATT,

PCAF#3: GGCUACGUCCAGGAGCGCACC,

YAP#1: GACAUCUUCUGGUCAGAGA,

YAP#2: CUGGUCAGAGAUACUUCUU.

### 2.7. SA*β*G Staining for Cells and Tissues

Sa*β*G staining (Beyotime, C0602, Shanghai, China) of HUVECs, aorta, and frozen tissue sections was performed according to the manufactures' instructions. Sa*β*G staining activity was measured as previously described [17].

### 2.8. Immunohistochemistry

The aorta arch was embedded with in paraffin as described above. The paraffin block then dissected into 4 *μ*M thickness. Paraffin sections underwent deparaffinization, rehydration, and exposure to alkaline phosphatase block buffer for heat-induced epitope retrieval. Next, the sections were incubated in 0.3% hydrogen peroxide for 30 minutes, followed by incubation with 5% BSA for another 30 minutes, and primary antibody at 4°C overnight (anti-PCAF, anti-YAP, 1 : 200). The next day, sections were incubated with the corresponding second antibodies (ZSGB-BIO, Beijing, China) and counterstained with hematoxylin.

### 2.9. mRNA Decay Assays

The stability of mRNA was performed by treating cells with Actinomycin D (100 *μ*g/mL) (MCE, HY-17559, Shanghai, China), a general RNA polymerase inhibitor. After 48 h incubation with PA, cells were treated with Actinomycin D for 0, 6, and 12 hours. Then mRNA levels were measured by qPCR.

### 2.10. Stability of Protein

To measure the effect of PA treatment on regulating the stability of PCAF protein, control and PA-stimulated HUVECs for 48 h before being treated with 35 *μ*mol/L cycloheximide (CHX) (MCE, HY-12320, Shanghai, China) for 0, 6, and 12 h or MG132 (10 *μ*M) for 0, 4, and 8 h. The total protein was extracted from whole-cell lysates and then prepared for western blotting analysis.

### 2.11. Co-Immunoprecipitation

Cells were transfected with corresponding plasmids (Addgene, #8941, #13054, #17793) and lysed in co-IP buffer containing protease inhibitor cocktail tablets. The indicated Flag-tagged magnetic beads or His-tagged beads (Bimake, Shanghai, China) were incubated with cell lysates at 4°C overnight. Next day, the lysates were removed to 1.5 ml tube to detect if targeted proteins were fully combined with the magnetic beads and PBST was used to wash the beads for 5 minutes three times. Then, 1 × SDS were added for boiling. The immunocomplexes were subjected to western blotting using the indicated antibodies.

### 2.12. Biochemistry and Enzyme-Linked Immunosorbent Assay (ELISA)

Serum samples were collected from 4w, aged+AAV9-Luc and aged+AAV9-PCAF mice. Total IL-1*β* was determined by mouse IL-1*β* ELISA kits (R&D systems, Cat #PMLB00C, USA) following the manufacturer's instructions [18].

### 2.13. Pathway Enrichment Analysis

The data were downloaded from GEO database (GSE47179). Kyoto Encyclopedia of Genes and Genomes (KEGG) pathway analysis was used to determine the associated biological pathways after PCAF silence. In addition, the DAVID online tool was applied to KEGG pathway analysis [19, 20]. A *P* value of <0.05 was considered significant.

### 2.14. Statistical Analysis

At least 6 biological replicates and 3 replicates were done for each experiment. Results are presented as the means ± standard deviations (SDs). Student-*t* test or one-way ANOVA analysis were used for statistical analysis where appropriate.

## 3. Results

### 3.1. PCAF Was Increased in Aged Vascular Tissues and HUVECs Underwent X Radiation or Palmitate Acid Treatment

**Figure 1:**
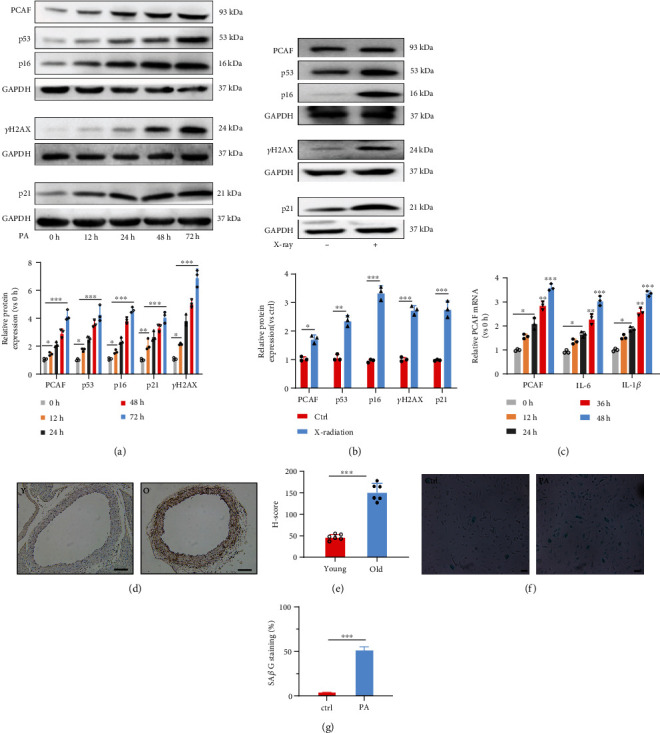
Increased PCAF expression in endothelia of aged mice and palmitate acid (PA) or X radiation treated HUVECs. (a) Western blotting analysis and qualification of PCAF and senescence-associated protein expression in HUVECs stimulated with PA (0.3 mM) for the indicated time points. (b) Western blotting analysis and qualification of PCAF and senescence-associated protein expression in HUVECs treated with or without X radiation (15 gray). (c) mRNA levels of PCAF, IL-6, and IL-1*β* in HUVECs treated with vehicle or PA for indicated time points. (d) and (e) Immunohistochemical (IHC) stain and quantitative analysis of blood vessels from young (4 w) and old (one and half-year-old) mice with PCAF antibody. Scale bars: 100 *μ*m. (f) and (g) Sa*β*G staining and quantitative analysis of vector and PA-treated HUVECs. Scale bars, 20 *μ*m. Y: young mice; O: old mice. Data are expressed as mean ± SEM (^∗^*P* < 0.05, ^∗∗^*P* < 0.01, ^∗∗∗^*P* < 0.001, n.s. *P* > 0.05).

To explore the role of PCAF in endothelial senescence, we first investigated PCAF expression in vascular vessels between young (4 w) and aged mice (one and half-year-old). Immunohistochemical (IHC) analysis revealed that PCAF expression was significantly upregulated in the aortic endothelia of aged mice (Figure 1(d)). Then, we examined PCAF mRNA and protein expression in actively dividing young HUVECs (defined as cells used before passage 5), young HUVECs underwent palmitate acid (PA) treatment or X radiation (15 gray). HUVECs developed stress-induced premature senescence after X radiation and PA treatment featured by reduced proliferation and increased Sa*β*G staining activity (Figure 1(f)), remarkable upregulation of senescence-associated p53, p21, P16 gene, and DNA damage marker pH2AX (a phosphorylated form of the histone variant H2AX, which is increased in DNA damage response) (Figures 1(a) and 1(b)) [21]. This demonstrated that PA treatment can imitate cellular senescence induced by X radiation. So, in this study, PA treatment was used to induce cellular senescence. Meanwhile, Western blotting showed that PCAF expression was increased in PA-treated/X radiated cells than the control young cells (Figures 1(a) and 1(b)). In addition, palmitate or X radiation promoted IL-6 and IL-1*β* mRNA expression in young HUVECs (Figure 1(c)). These results suggested PCAF may participate in the process of cellular senescence.

### 3.2. PCAF Regulated PA-Induced Cellular Senescence in HUVECs

**Figure 2:**
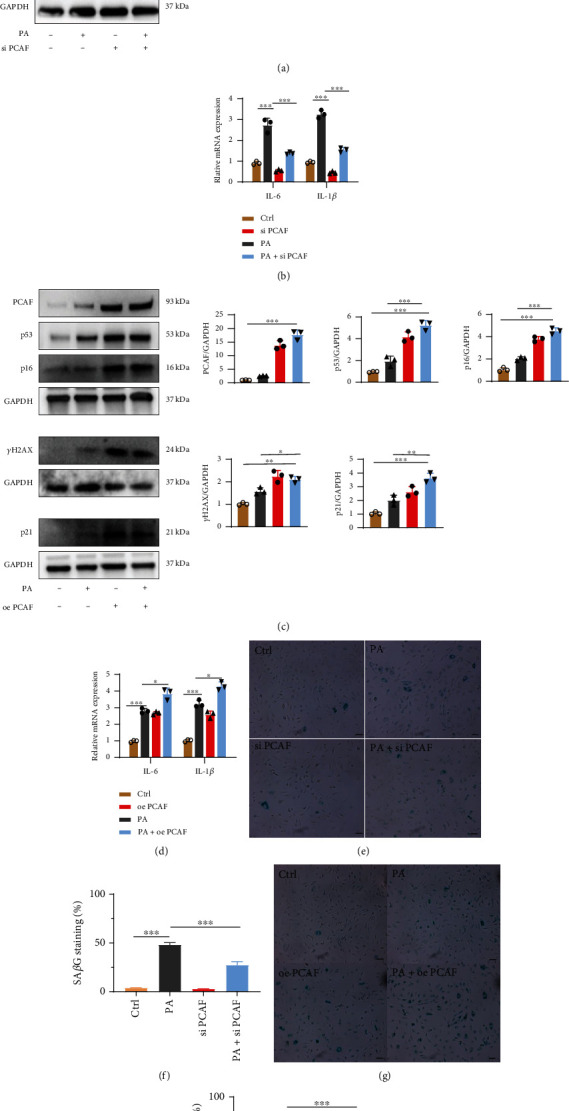
PCAF were involved in the regulation of endothelial senescence. (a) and (b) Western blotting analysis and qualification of p53, p16, pH2A.X, and p21 protein expression and relative mRNA expression of IL-6 and IL-1*β* in PA-treated HUVECs transfected with scramble or PCAF siRNA. (c) and (d) Western blotting analysis and qualification of p53, p16, pH2A.X, and p21 protein expression and relative mRNA expression of IL-6 and IL-1*β* in PA-treated HUVECs transfected with vector or PCAF plasmid. (e) and (f) Sa*β*G staining and quantitative analysis in HUVECs transfected with scramble or PCAF siRNA in the presence of PA or not. Scale bars, 20 *μ*m. (g) and (h) Sa*β*G staining and quantitative analysis in HUVECs transfected with vector or PCAF plasmid in the presence of PA or not. Scale bars, 20 *μ*m. Data are expressed as mean ± SEM (*n* = 3 for each experiment. (^∗^*P* < 0.05, ^∗∗^*P* < 0.01, ^∗∗∗^*P* < 0.001, n.s. *P* > 0.05).

We next investigated whether PCAF promoted PA-induced cellular senescence in HUVECs. First, silencing of PCAF significantly decreased the levels of p53, p21, p16, and pH2AX in PA-stimulated HUVECs (Figure 2(a)). PCR results also showed that inhibition of PCAF in HUVECs decreased PA-induced upregulation of IL-1*β* and IL-6 mRNA levels (Figure 2(b)). Moreover, the silence of PCAF decreased Sa*β*G staining in PA-stimulated HUVECs (Figure 2(e)), while overexpression of PCAF in PA-stimulated HUVECs upregulated the levels of p53, p21, p16, and pH2A.X protein levels, IL-1*β* and IL-6 mRNA and Sa*β*G staining (Figures 2(c), 2(d), and 2(f)). Taken together, these gain and loss-of-function experiments suggested that PCAF mediated PA-induced endothelial senescence.

### 3.3. PA Upregulated PCAF in HUVECs Depended on Decreased MDM2 Mediated Ubiquitination

**Figure 3:**
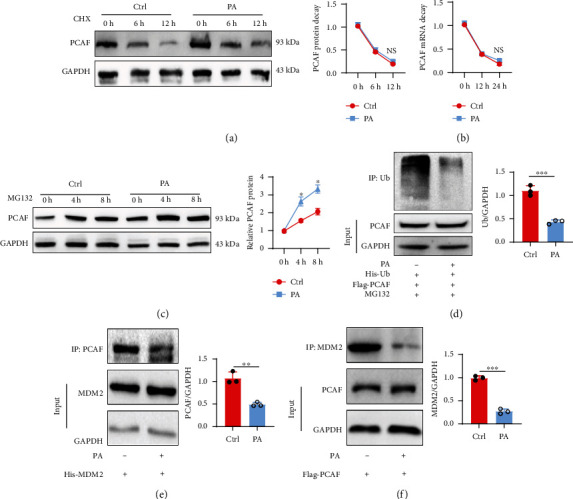
PA upregulated PCAF in HUVECs depended on decreased MDM2 mediated ubiquitination. (a) HUVECs were treated with cycloheximide (35 *μ*mol/L) to block nascent protein synthesis for indicated time points in the presence of PA or not. Stability of PCAF protein was assessed by western blotting. (b) The stability of PCAF mRNA was assessed in HUVECs treated with actinomycin D (100 *μ*g/ml) by RT-PCR in the presence of PA or not. (c) Western blotting was used to assess the expression of PCAF in HUVECs treated with MG132 (10 *μ*M) for indicated times points in the presence of PA or not. (d) His-tagged ubiquitin plasmid and flag-tagged PCAF plasmid were cotransfected into HUVECs, PBS, or PA were then stimulated for 48 h. 12 h before cells were harvested, MG132 was added into the culture dishes. Flag-tagged magnetic beads were used to bind to PCAF, total Ub level was detected by western blotting. (e) and (f) His-tagged MDM2 plasmid or Flag-tagged PCAF plasmid was transfected into HUVECs before stimulated with PBS or PA for 48 h, Co-IP were performed by using corresponding magnetic beads. Data are expressed as mean ± SEM (*n* = 3 for each experiment. ^∗^*P* < 0.05, ^∗∗^*P* < 0.01, ^∗∗∗^*P* < 0.001, n.s. *P* > 0.05).

Further, we determined the potential mechanism of PA-stimulated PCAF expression. We investigated if PA stimulation influenced PCAF mRNA or protein stability in HUVECs. HUVECs were stimulated with PA before treatment with actinomycin D (100 *μ*g/ml) or not. Our results revealed that PA stimulation did not affect PCAF mRNA degradation (Figure 3(b)). Meanwhile the half-life of PCAF protein was also explored after PA treatment or not. PA treatment increased PCAF protein stability (Figures 3(a), 3(c)). Then we tested whether PA increased PCAF protein stability via reduced ubiquitination. HUVECs were transfected with His-tagged ubiquitin plasmid in the presence of PA or not for 48 hours, MG132 then was added 12 h before cells were harvested. Ubiquitin was co-immunoprecipitated by His-tagged magnetic beads. Co-IP showed PA stimulation evidently reduced overall ubiquitination level. Interestingly, previous studies have documented that MDM2 is a nuclear-localized E3 ubiquitin ligase that mediates the ubiquitination of many proteins including PCAF [22, 23]. Thus, we validated the potential interaction between PCAF and MDM2. Co-immunoprecipitation (Co-IP) experiments revealed that compared with control group, PA stimulation decreased the interaction between PCAF and MDM2 (Figures 3(e) and 3(f)). To sum up, PA-stimulated PCAF upregulation was dependent on decreased MDM2 mediated ubiquitination.

### 3.4. PCAF Mediated PA-Induced Endothelial Senescence through Hippo Signaling Pathway

**Figure 4:**
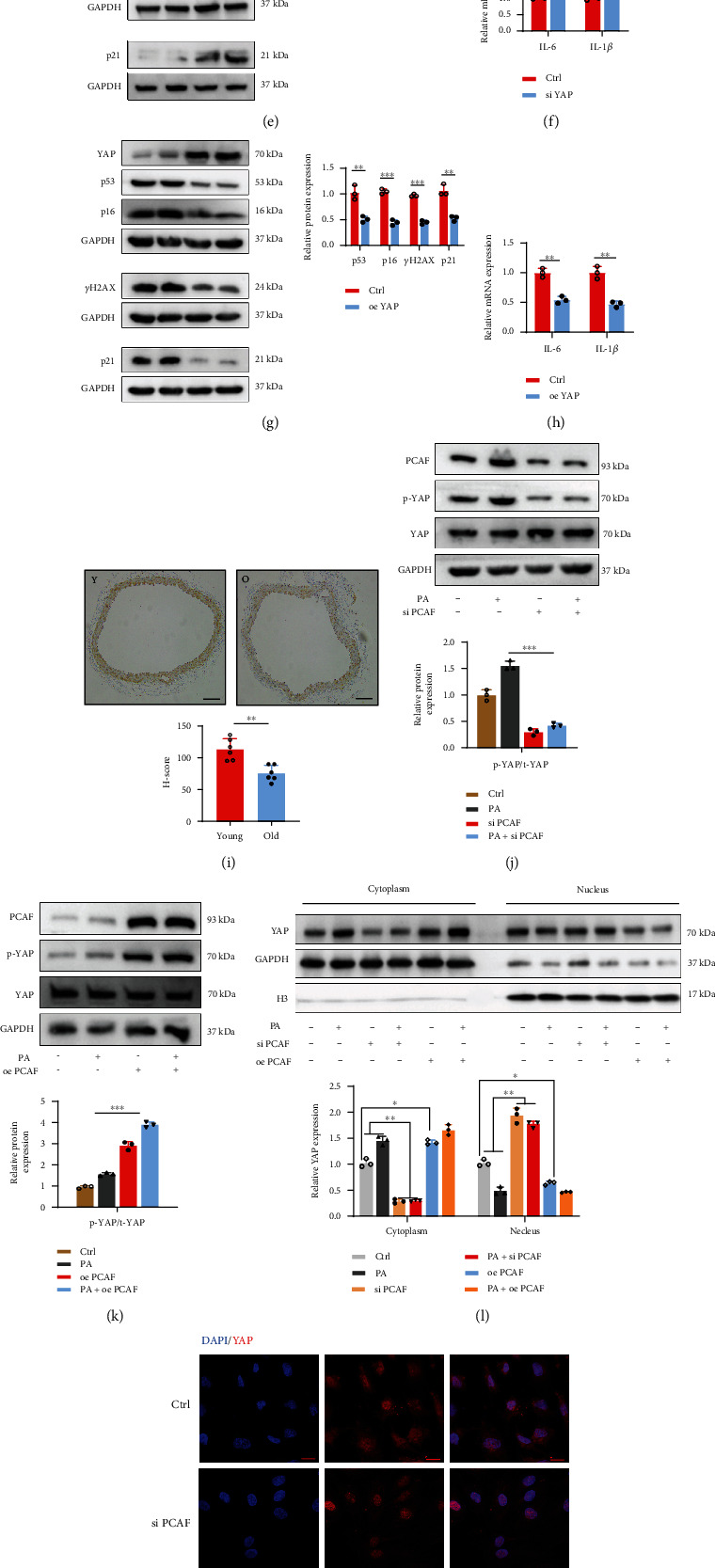
PCAF mediated PA-induced endothelial senescence through Hippo signaling pathway. (a) KEGG analysis showed that PCAF may regulate endothelial senescence via Hippo-YAP pathway. (b) Western blotting analysis and qualification of phosphorylated YAP expression in HUVECs treated with PA for the indicated time points. (c) Immunofluorescence of YAP localization in HUVECs treated with or without PA. Scar bars, 10 *μ*m. (d) Expression of total YAP proteins was analyzed by immunoblot in nuclear and cytoplasmic protein extractions from in HUVECs treated with or without PA. (e) and (f): HUVECs were transfected with YAP siRNA or overexpression plasmid. The expression of p53, p16, pH2A.X and p21 protein were by immunoblotting. (g) and (h) HUVECs were transfected with YAP siRNA or overexpression plasmid, IL-6 and IL-1*β* mRNA expression were analyzed by RT-PCR. (i) IHC staining and its qualification of vessels from 4w and one and half-year-old mice with YAP antibody. Scale bars, 100 *μ*m. (j) and (k) HUVECs were transfected with PCAF siRNA or overexpression plasmid, the expression of p-YAP and t-YAP protein were by immunoblotting. (l) HUVECs were transfected with PCAF siRNA or overexpression plasmid treated with or without PA. Expression of YAP proteins was analyzed by immunoblot in nuclear and cytoplasmic protein extractions. (m) Immunofluorescence of YAP localization in HUVECs infected with si PCAF or PCAF plasmid or vector. Scar bars, 10 *μ*m. (n) and (o) Flag-tagged YAP or Flag-tagged PCAF was transfected into HUVECs before stimulated with PBS or PA for 48 h, Co-IP were performed by using corresponding magnetic beads. t-YAP: total YAP; p-YAP: phosphorylated YAP. Data are expressed as mean ± SEM (*n* = 3 for each experiment. ^∗^*P* < 0.05, ^∗∗^*P* < 0.01, ^∗∗∗^*P* < 0.001, n.s. *P* > 0.05).

To further understand the molecular mechanism by which PCAF modulated endothelial senescence, we predicted the potential pathways PCAF may participate in by using Gene Ontology annotation and predicted that PCAF may mainly regulate endothelial senescence via endocytosis and the Hippo signaling pathway (Figure 4(a)). Clathrin-mediated endocytosis (CME) and caveolae-mediated endocytosis (CavME) represent major types of endocytosis that are implicated in senescence [24]. Knockdown of PCAF had no significant effect on clathrin and caveolae protein expression, indicating PCAF-mediated endothelial senescence may not influence endocytosis (Suppl. Figure 2C). YAP plays a central role in the Hippo signaling pathway [25]. So, we tested whether PCAF regulated endothelial senescence via Hippo-YAP pathway. Western blotting and immunofluorescence revealed that PA stimulation reduced the transportation of YAP into the nucleus (Figures 4(c) and 4(d)). In addition, the phosphorylation of YAP was upregulated upon PA treatment (Figure 4(b)). Then, we knocked down YAP using siRNA in HUVECs, silence of YAP significantly increased the expression of p53, p21, p16, and pH2AX (Figure 4(e)). Real-time PCR revealed that YAP knockdown upregulated the mRNA levels of IL-1*β* and IL-6 (Figure 4(f)). In addition, YAP overexpression in HUVECs using plasmid prevented these senescence-associated gene expression and the mRNA levels of IL-1*β* and IL-6 (Figures 4(g) and 4(h)). IHC analysis also revealed that there was less expression of YAP in the vascular endothelia from aged mice than the control group (Figure 4(i)). To investigate if PCAF regulated cellular senescence through Hippo signaling pathway, we knocked down PCAF in HUVECs. Our results showed PACF silence decreased YAP phosphorylation and its nucleus exporting (Figures 4(j), 4(m)). Accordingly, overexpression of PCAF in HUVECs promoted YAP phosphorylation and its transportation to the cytoplasm (Figures 4(k) and 4(l)). Additionally, PCAF knockdown or overexpression can also influence the activation of PDZ-binding motif (TAZ; also known as WWTR1) (Suppl. Figures 2A and 2B), which often acts as a coactivator with YAP to regulate various cellular process. Co-IP results showed that PCAF can bind to YAP and TAZ in HUVECs and their binding increased when PA stimulated (Figures 4(n) and 4(o)). In all, these results indicated that PCAF may regulate endothelial senescence via the Hippo signaling pathway.

### 3.5. YAP Is the Downstream Target of PCAF in PA-Stimulated Senescence-Associated Phenotypes in HUVECs

**Figure 5:**
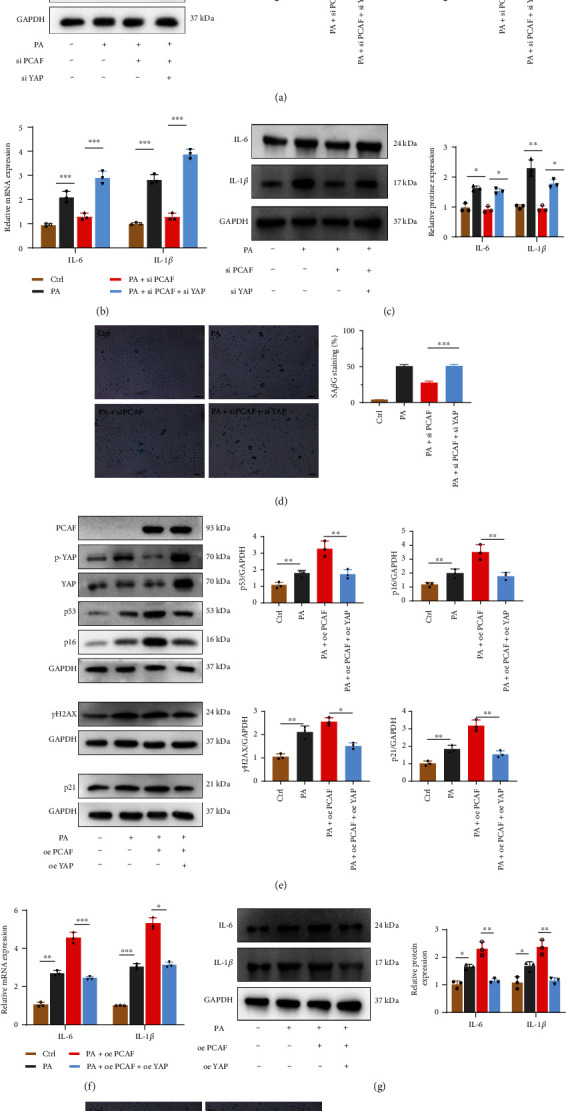
YAP is the downstream of PCAF in PA-stimulated senescence in HUVECs. (a)–(c) PCAF and YAP were knocked down in HUVECs using siRNA in the treatment of PA or not. The expression of p53, p16, pH2A.X, and p21 protein were by immunoblotting (a), relative IL-6 and IL-1*β* mRNA expression were analyzed by RT-PCR (b) and immunoblotting (c). (d) Quantitative analysis of Sa*β*G staining. Scale bars, 20 *μ*m. E-G: PCAF and YAP were overexpressed in HUVECs using plasmids in the treatment of PA or not. The expression of p53, p16, pH2A.X, and p21 protein were by immunoblotting (e), relative IL-6 and IL-1*β* mRNA expression were analyzed by RT-PCR (f) and immunoblotting (g). (h) Quantitative analysis of Sa*β*G staining. Scale bars, 20 *μ*m. Data are expressed as mean ± SEM (*n* = 3 for each experiment. ^∗^*P* < 0.05, ^∗∗^*P* < 0.01, ^∗∗∗^*P* < 0.001, n.s. *P* > 0.05).

To confirm that YAP was the downstream of PCAF in PA-stimulated cellular senescence, PCAF knockdown significantly reduced PA-induced the upregulation of p53, p21, and p16 expression, and YAP silence remarkably reversed the reduced expression of these senescence-associated proteins (Figure 5(a)). As compared with silence control, YAP silence reversed PCAF-silencing-reduced Sa*β*G staining in PA-treated HUVECs (Figure 5(d)). The mRNA and protein levels of IL-1*β* and IL-6 expression showed the similar trend (Figures 5(c) and 5(c)). Then, we forced the expression of PCAF and YAP in HUVECs. Compared with vector plasmid, the overexpression of YAP significantly abolished PCAF-induced senescence-associated phenotypes in HUVECs (Figures 5(e), 5(h)). Similarly, the upregulation of YAP also reduced the increased mRNA and protein levels of IL-1*β* and IL-6 mediated by PCAF in PA-treated HUVECs (Figures 5(f) and 5(g)). To sum up, this demonstrated that YAP is the downstream of PCAF in PA-induced cellular senescence.

### 3.6. Knockdown of PCAF Ameliorated Vascular Senescence

**Figure 6:**
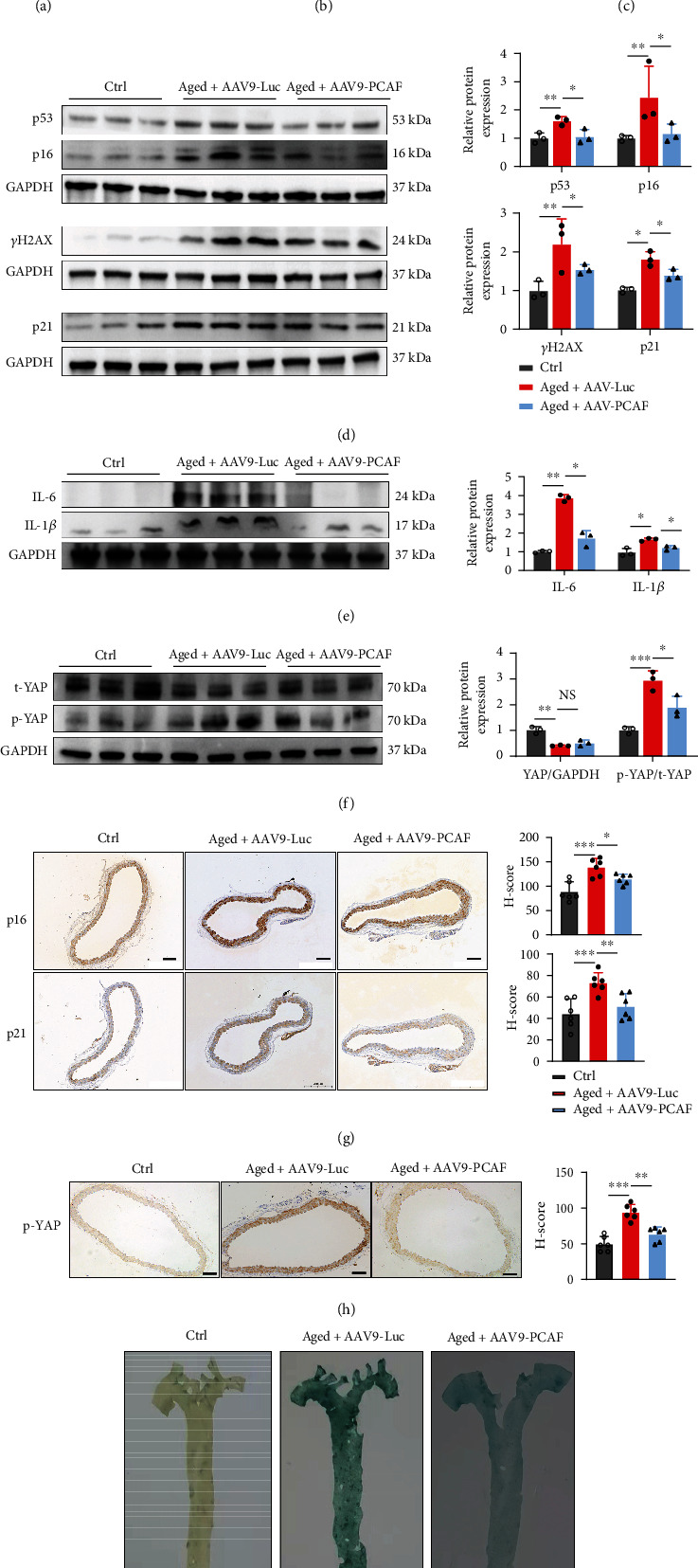
Knockdown of PCAF ameliorated vascular senescence. (a) The concentration of IL-1*β* in the plasma from ctrl (*n* = 6), aged+AAV9-Luc (*n* = 8), and aged+AAV9-PCAF (*n* = 7) mice were determined by ELISA kit according to its instruction. (b) and (c) Representative Sa*β*G staining of aortic sinus sections (b) and its qualification analysis and thoracoabdominal aorta (i). Scale bars, 20 *μ*m. (d) and (e): The expression of senescence-associated markers (d) and inflammatory factors (e) in the aorta from each group were assessed by immunoblotting. (f) The expression of YAP and p-YAP were investigated by immunoblotting. (g) and (h) The expression of p16, p21 (g) and p-YAP (h) were detected by IHC. Scale bars, 20 *μ*m. Data are expressed as mean ± SEM (^∗^*P* < 0.05, ^∗∗^*P* < 0.01, ^∗∗∗^*P* < 0.001, n.s. *P* > 0.05).

In PA-stimulated HUVECs, PCAF overexpression significantly accelerated cellular senescence and its downregulation ameliorated cellular senescence. Therefore, we tested if PCAF EC-specific downregulation using adeno-associated virus (AAV) can ameliorate vascular endothelial senescence in vivo. One and half-year-old C57BL6 male mice were injected with AAV-tie-PCAF virus or control virus and then fed with rodent diet for 16 weeks. Primary endothelial cells were isolated and vascular senescence-associated phenotypes and inflammatory factors were investigated by western blotting (Suppl. Figures 2E and 2F). Circulated IL-1*β* concentration in plasma was slightly reduced in PCAF-knockdown mice but with no significance (Figure 6(a)). No significance in body weight, blood glucose, serum triglyceride, total cholesterol levels, LDL, and HDL were found between the two groups of aged mice (Suppl. Figures 1E and 1F). Western blotting analysis suggested that senescence-associated markers were downregulated in the aorta of PCAF-knockdown mice (Figure 6(d) and 6(e)). The level of p-YAP/YAP was upregulated in the aorta of aged mice and downregulated in endothelial PCAF-knockdown mice (Figure 6(f)). IHC analysis of p16 and p21 and p-YAP revealed the same trend. (Figure 6(g) and 6(h)). There was a remarkable reduction of Sa*β*G staining of aortas and frozen tissue sections of aortic sinus in PCAF-knockdown mice than the control aged mice (Figure 6(b), 6(i)). These in vivo experiments suggested that PCAF might aggravate vascular senescence.

## 4. Discussion

Cellular senescence is a continuously irreversible process that chronically leads to organ dysfunction. The way to slow down this process at a systemic or tissue level has attracted much concern over the past decades. Cardiovascular disease, the most common cause of death, is also age-related disease [26]. Aged vascular tissues can bring about a series of diseases including atherosclerosis and vascular calcification [27, 28]. Thus, it is important to understand the mechanisms behind cellular senescence.

**Figure 7:**
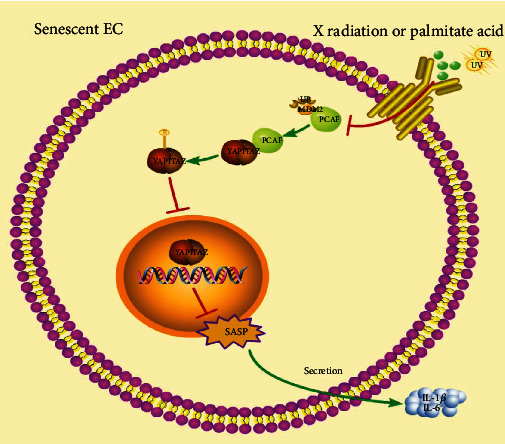
A proposed model for the role of PCAF in endothelial senescence. PCAF is upregulated in senescent endothelial cells by decreased coupling with DMD2 to inhibit the transportation of YAP/TAZ into nucleus, thereby promoting cellular senescence.

PCAF, a member of the GCN5-related N-acetyltransferase family with histone acetyltransferase activity, has been shown to be involved in cell growth and differentiation, tumorigenesis, and transcriptional regulation [8]. Prior works show that PCAF can regulate the activation of p53/p21 and Rb/p16 signaling pathway. PCAF downregulation can also inhibit NF-*κ*B-mediated vascular inflammation, which is a critical cause of cellular senescence [29]. These results have put PCAF in the spotlight of the aging process. However, the specific role of PCAF in regulating cellular senescence is unclear. In this study, we found that (a) PCAF was highly expressed in aged vascular artery and its expression was closely related with the upregulation senescence-associated phenotype markers and inflammatory factors in palmitate acid-induced aged HUVECs, (b) genetical silence of PCAF significantly attenuated cellular senescence-associated phenotypes in vivo, and (c) PCAF regulated cellular senescence via the Hippo signaling pathway. The possible mechanism of this study is summarized in Figure 7.

In our preliminary study, we found that PCAF could be activated by PA or UV stimulation in HUVECs, along with increased senescence-associated markers like p53, p21, p16, and DNA damage marker pH2AX. Knockdown of PCAF in PA-stimulated HUVECs alleviated senescence-associated phenotypes and overexpression of PCAF accelerated it. Excitingly, in aged mice injected with AAV-tie-PCAF virus, Sa*β*G staining showed PCAF downregulation in endothelial cells obviously alleviated vascular senescence. Therefore, our results indicated that PCAF can affect cellular senescence and the key role of PCAF in this process may provide a therapeutic target for the prevention of senescence-associated vascular diseases.

Based on transcriptional microarray analysis, Kyoto Encyclopedia of Genes and Genomes pathway enrichment analysis showed that genes were mainly enriched in endocytosis and the Hippo signaling pathway. Our results indicated that PCAF might induce cellular senescence via the Hippo signaling pathway without affecting endocytosis in HUVECs. Studies over the past decade have uncovered the critical role of the Hippo-YAP signaling pathway in the regulation of development, regeneration, and homoeostasis [30, 31]. When the Hippo pathway is active, the Hippo kinase cascade phosphorylates YAP and TAZ, resulting in their cytoplasmic retention and proteolytic degradation. When the Hippo pathway is inactive, YAP and TAZ translocate into the nucleus and interact with transcription factors to regulate the expression of target genes. Previous works also manifested the effectiveness of YAP and TAZ in cellular senescence [32]. However, it seems that in various types of cells, the effects of YAP in modulating cellular senescence are quite different. For instance, downregulation of YAP in IMR90 tumor cells increased cellular senescence, whereas upregulation of YAP in Werner syndrome-derived fibroblasts accelerated cellular senescence [33, 34]. Recent work indicated that the upregulation of YAP may regulate cellular senescence through YAP-mTOR-autophagic flux signaling pathway [35].

In our study, we found that PCAF-induced cellular senescence was repressed by YAP overexpression. PCAF upregulated the phosphorylation of YAP and TAZ in PA-induced senescent HUVECs while not apparently affecting the mTOR pathway, which was documented to have a direct link with the Hippo signaling pathway and play an important role in cellular senescence (Suppl. Figure 2D). PCAF silence promoted YAP and TAZ nucleus transportation, result in lessened senescence-associated secretory phenotype (SASP) and reduced IL-1*β* and IL-6 production. Regarding to how PCAF affecting YAP and TAZ activity, our Co-IP results revealed that PCAF can bind to YAP and TAZ in HUVECs, implying PCAF may directly regulate the activation YAP and TAZ, result in increased phosphorylation of YAP and TAZ. However, the specific mechanism between PCAF and YAP/TAZ needs further study. In addition to our in vitro study for the modulation of PCAF in cellular senescence, we demonstrated that PCAF increased in the endothelia of aorta in aged mouse and EC-specific knockdown of PCAF evidently neutralized vascular senescence. The limitation of this study is that mice injected with adeno-associated virus (AAV)-tie-YAP are not included in this study to further verify our hypothesis in vivo.

In conclusion, our study revealed a previously unknown, but potentially crucial role of PCAF in regulating endothelial cell senescence. We propose that inhibition the PCAF in endothelial cell might provide a novel strategy for prevention and treatment of senescence-associated vascular disease.

## 5. Conclusions

Our study indicated that PCAF was closely related to vascular senescence. Its upregulation in vascular tissue accelerated vascular senescence and PCAF inhibition effectively alleviated vascular senescence. In addition, YAP inactivation was observed during PCAF-promoted vascular senescence. These findings indicated a fundamentally important function of PCAF as a potent target for controlling endothelial cell senescence. We propose that inhibition of PCAF might provide a novel strategy for prevention and treatment of vascular senescence.

## Data Availability

The data used for pathway enrichment analysis were downloaded from GEO database (GSE47179).

## Supplementary Materials

Supplementary Materials Supplemental Figure 1–A and B: PCAF and YAP siRNA efficiency was tested via western blotting in HUVECs. C and D: Body weight, serum triglyceride, total cholesterol, LDL and HDL levels of each group are shown. Supplemental Figure 2–A and B: Western blotting analysis and qualification of p-TAZ and TAZ in PA-treated HUVECs transfected with scramble or PCAF siRNA. C: Western blotting analysis and qualification of clathrin and caveolin in HUVECs transfected with scramble or PCAF siRNA. D: Western blotting analysis and qualification of p-mTOR and mTOR in HUVECs transfected with scramble or PCAF siRNA. E and F: Primary endothelial cells were isolated and vascular senescence-associated phenotypes (E) and inflammatory factors (F) were investigated by western blotting. Physiological parameters of experimental mice.
