# Supplementary material for: PCAF Accelerates Vascular Senescence via the Hippo Signaling Pathway
Source: Oxid Med Cell Longev. 2022 Oct 6;2022:1200602. doi: 10.1155/2022/1200602 (PMC9560818; doi:10.1155/2022/1200602)
Supplement: Supplementary Materials — Supplemental Figure 1–A and B: PCAF and YAP siRNA efficiency was tested via western blotting in HUVECs. C and D: Body weight, serum triglyceride, total cholesterol, LDL and HDL levels of each group are shown. Supplemental Figure 2–A and B: Western blotting analysis and qualification of p-TAZ and TAZ in PA-treated HUVECs transfected with scramble or PCAF siRNA. C: Western blotting analysis and qualification of clathrin and caveolin in HUVECs transfected with scramble or PCAF siRNA. D: Western blotting analysis and qualification of p-mTOR and mTOR in HUVECs transfected with scramble or PCAF siRNA. E and F: Primary endothelial cells were isolated and vascular senescence-associated phenotypes (E) and inflammatory factors (F) were investigated by western blotting. Physiological parameters of experimental mice. [file 1200602.f1.docx]

**Supplementary Materials:**


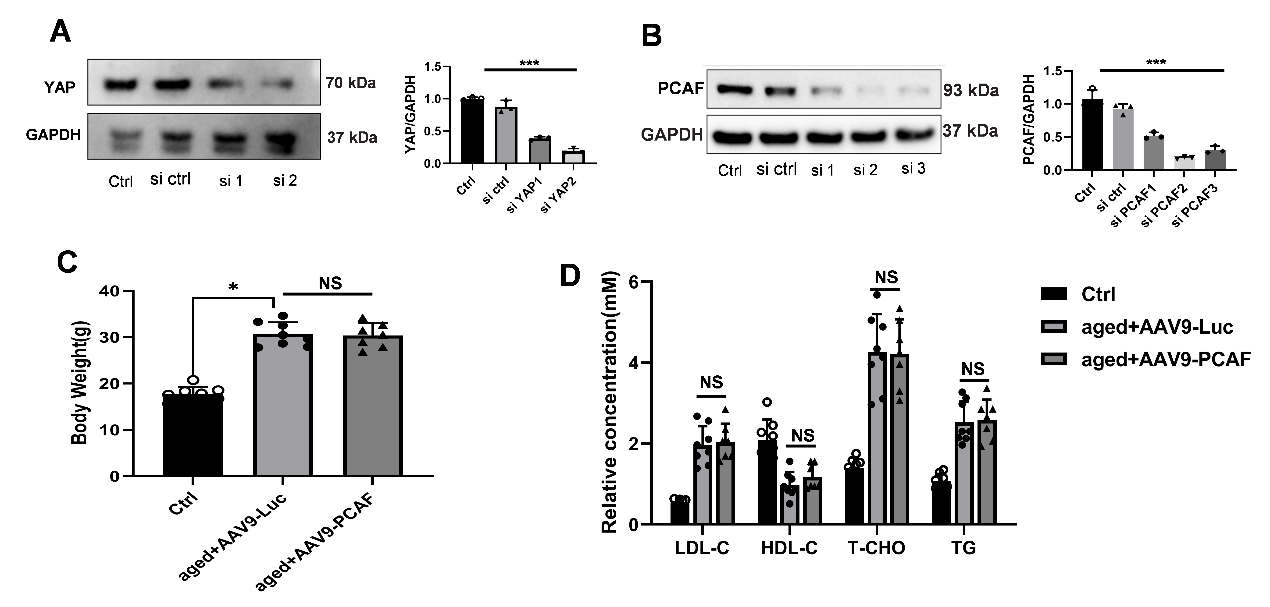
 **Supplemental Figure1** **–** A and B: PCAF and YAP siRNA efficiency was tested via western blotting in HUVECs. C and D: Body weight, serum triglyceride, total cholesterol, LDL and HDL levels of each group are shown.


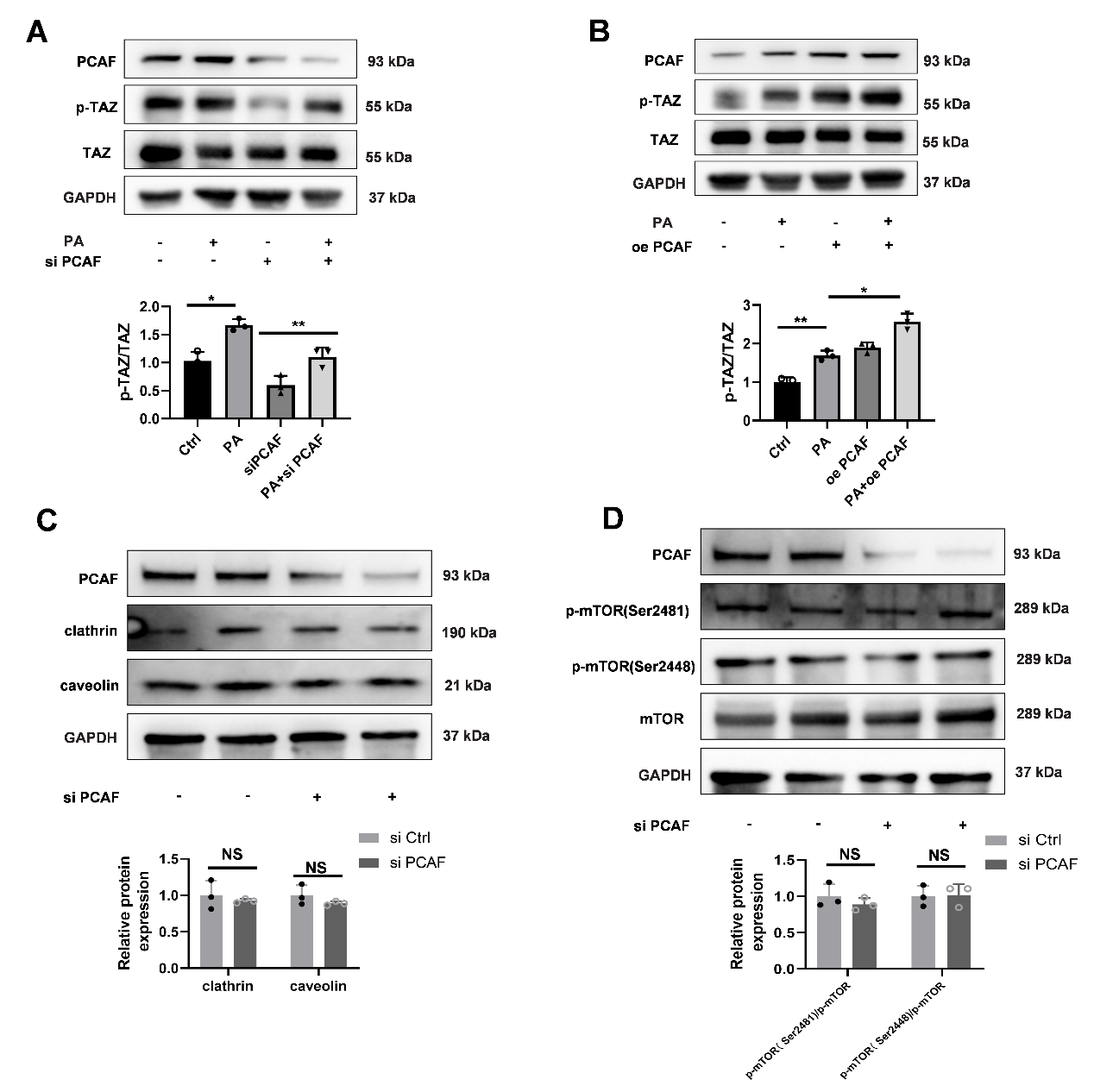


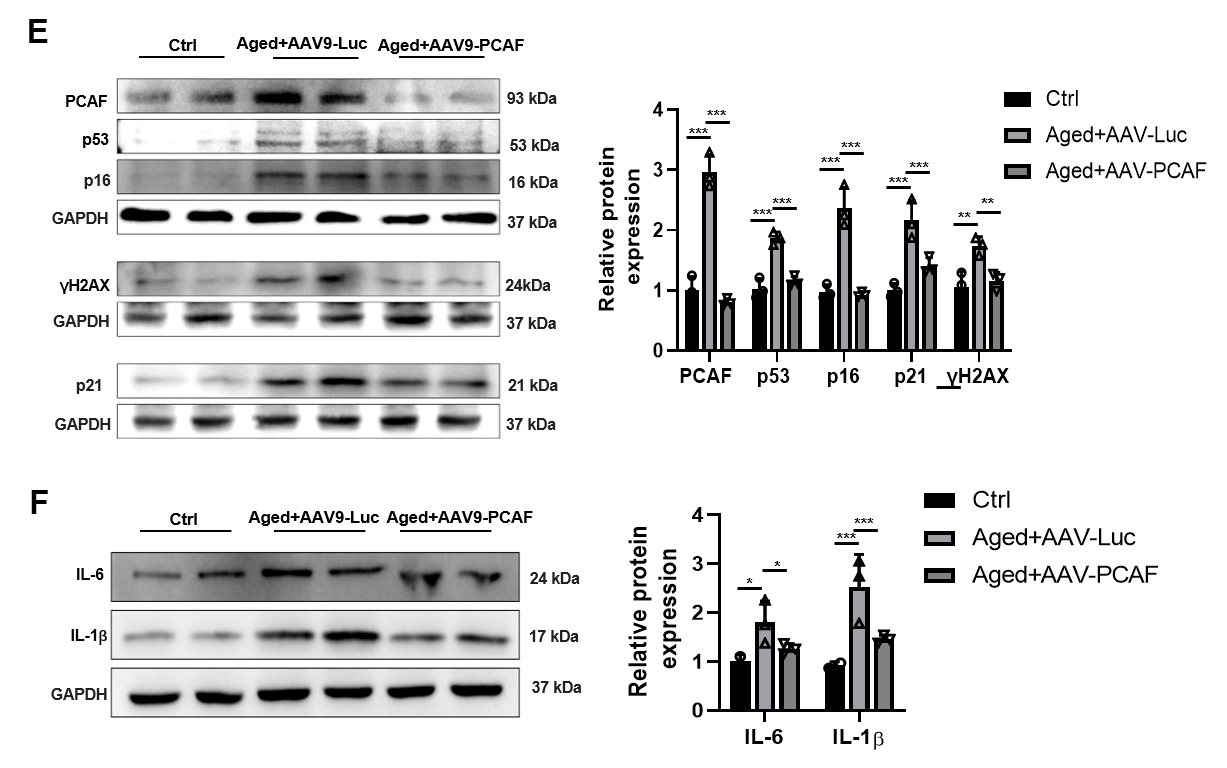


**Supplemental Figure2 –** A and B: Western blotting analysis and qualification of p-TAZ and TAZ in PA treated HUVECs transfected with scramble or PCAF siRNA. C: Western blotting analysis and qualification of clathrin and caveolin in HUVECs transfected with scramble or PCAF siRNA. D: Western blotting analysis and qualification of p-mTOR and m-TOR in HUVECs transfected with scramble or PCAF siRNA. E and F: Primary endothelial cells were isolated and vascular senescence-associated phenotypes (E) and inflammatory factors (F) were investigated by western blotting.

**Physiological parameters of experimental mice.**

| **Parameters** | **Control** | **Aged+AAV9-Luc** | **Aged+AAV9-PCAF** |
| --- | --- | --- | --- |
| Body weight (g) | 17.66±1.631 | 30.615±2.595^*^ | 30.381±2.647 |
| Liver weight (g) | 1.205±1.537 | 1.881±2.032^*^ | 1.823±2.067 |
| Spleen weight (mg) | 153.3±3.076 | 180.4±5.632^*^ | 179±5.037 |
| Heart weight (mg) | 135.4±2.526 | 150.3±2.372^*^ | 149.2±3.342 |
| Kidney weight (mg) | 327.3±2.739 | 360.3±3.203^*^ | 372.1±2.973^#^ |
| Blood glucose (mM) | 7.36±0.973 | 6.963±1.028 | 7.108±0.873 |

Note: Control, n=6; Aged+AAV9-Luc, n=8; Aged+AAV9-PCAF, n=7 (one mouse from Aged+AAV9-PCAF group died before harvest). Data are shown by mean ± SD. ^*^*P<*0.05 vs. Control; ^#^*P*<0.05 vs. Aged+AAV9-Luc.
